# *Aedes albopictus* and Its Environmental Limits in Europe

**DOI:** 10.1371/journal.pone.0162116

**Published:** 2016-09-07

**Authors:** Sarah Cunze, Judith Kochmann, Lisa K. Koch, Sven Klimpel

**Affiliations:** 1 Goethe-University, Institute for Ecology, Evolution and Diversity; Max-von-Laue-Str. 13, D-60438 Frankfurt/ M., Germany; 2 Senckenberg Biodiversity and Climate Research Centre; Senckenberg Gesellschaft für Naturforschung; Senckenberganlage 25, D-60325 Frankfurt/ M., Germany; Universite Francois-Rabelais de Tours, FRANCE

## Abstract

The Asian tiger mosquito *Aedes albopictus*, native to South East Asia, is listed as one of the worst invasive vector species worldwide. In Europe the species is currently restricted to Southern Europe, but due to the ongoing climate change, *Ae*. *albopictus* is expected to expand its potential range further northwards. In addition to modelling the habitat suitability for *Ae*. *albopictus* under current and future climatic conditions in Europe by means of the maximum entropy approach, we here focused on the drivers of the habitat suitability prediction. We explored the most limiting factors for *Aedes albopictus* in Europe under current and future climatic conditions, a method which has been neglected in species distribution modelling so far. *Ae*. *albopictus* is one of the best-studied mosquito species, which allowed us to evaluate the applied Maxent approach for most limiting factor mapping. We identified three key limiting factors for *Ae*. *albopictus* in Europe under current climatic conditions: winter temperature in Eastern Europe, summer temperature in Southern Europe. Model findings were in good accordance with commonly known establishment thresholds in Europe based on climate chamber experiments and derived from the geographical distribution of the species. Under future climatic conditions low winter temperature were modelled to remain the most limiting factor in Eastern Europe, whereas in Central Europe annual mean temperature and summer temperatures were modelled to be replaced by summer precipitation, respectively, as most limiting factors. Changes in the climatic conditions in terms of the identified key limiting factors will be of great relevance regarding the invasive potential of the *Ae*. *albopictus*. Thus, our results may help to understand the key drivers of the suggested range expansion under climate change and may help to improve monitoring programmes. The applied approach of investigating limiting factors has proven to yield valuable results and may also provide valuable insights into the drivers of the prediction of current and future distribution of other species. This might be particularly interesting for other vector species that are of increasing public health concerns.

## Introduction

The Asian tiger mosquito *Aedes albopictus* (Skuse, 1894) is native to tropical and subtropical regions of southeast Asia, but has managed to spread to all continents except Antarctica today [1–3]. The species’ high ability to migrate is largely facilitated by global trade, tourism and the species’ wide breeding adaptability to various habitats [4]. Due to its rapid expansion and its vectorial capacity for multiple pathogens (among others Chikungunya and dengue [5,6], *Ae*. *albopictus* is ranked among the 100 worst invasive species in Europe and worldwide (Delivering Alien Invasive Species Inventories for Europe, www.europe-aliens.org; Global Invasive Species Database, www.iucn.org).

Records of first occurrence of *Ae*. *albopictus* in Europe date back to 1979 in Albania and to 1990 in Northern Italy, where it has been able to establish in subsequent years. Today, the species forms stable populations mainly in Northern and Central Italy but also other parts of the Mediterranean region [2]. *Aedes albopictus* is assumed to find increasingly suitable habitat conditions in Central Europe due to climate change and thus, will be presumably able to expand its European range further northwards [7,6]. However, in Europe, *Ae*. *albopictus* is exposed to different climatic conditions compared to its native range. Generally, the native range habitats, mainly tropical and subtropical forests, are characterized by rather constant climate conditions. This enables populations of *Ae*. *albopictus* to exist without overwintering strategies [8,9], whereas in Europe, *Ae*. *albopictus* is subjected to different climatic conditions. The production of eggs undergoing a winter diapause facilitated to a large extent the species’ establishment outside the native range [10,9].

As suggested by many authors, climatic thresholds determine whether the invasion process of *Ae*. *albopictus* in Europe will continue, finally resulting in a permanent establishment of the species in these temperate region. In Europe, these thresholds are currently based on climate chamber experiments or derived from their geographical distribution (e.g. [11]). For example, a winter temperature of more than 0°C on average, is thought to be necessary in order to enable egg overwintering [5,9]. As the species needs small aquatic habitats for egg deposition and breeding places, an annual precipitation of at least 500 mm has been proposed which ensures the maintenance of breeding places [1,2,9]. Another suggested threshold is a mean annual temperature over 11°C [9], which reflects the species’ adaptation to higher temperatures (e.g. [12]). Thus, a summer temperature of 25–30°C is regarded to be optimal [13,9] for the establishment in the non-native range.

The potential distribution of *Ae*. *albopictus* in Europe under current and future climatic conditions has been modelled in several previous studies by means of correlative approaches (e.g. [7,6]) as well as process based approaches (as a GIS overlay, e.g. [1]). These models broadly agree in projecting a potential northwards enlargement of area with suitable climatic conditions for *Ae*. *albopictus* in the face of projected climate change.

Apart from firstly modelling the habitat suitability for *Ae*. *albopictus* under current and future climatic conditions in Europe by means of the maximum entropy approach, we here also focused on the drivers of the habitat suitability prediction. We explored the most limiting factors for *Ae*. *albopictus* in Europe under current and future climatic conditions, a method which has been neglected in species distribution modelling so far. In order to evaluate the robustness of the model against the choice of environmental variables, we used different combinations of climatic factors thought to be especially relevant for the potential distribution within the non-native range (temperature, precipitation and photoperiod [9]). We then applied the Maxent limiting factor mapping tool described in [14] in order to determine the variable that limits the modelled habitat suitability the most at a certain location. This approach has rarely been applied in species distribution modelling (e.g. in [15]), although the investigations of limiting factors may provide valuable insights into the drivers of predictions of current and future distributions of species, especially for vector species that are of increasing public health concern. In species distribution modelling, the future climatic conditions used for projecting the potential future range of a species usually represent the average climatic conditions over several years. The investigation of climatic limiting factors might also enable assessing the impact of short-term oscillations, e.g. what do extreme events such as a hot and dry summer mean for the invasive potential of the species. According to [14] the approach also yields a basis for comparisons with physiological knowledge.

Exploring the climatic limiting factors may help to understand the key drivers of the suggested range expansion under future climate change and may at the same time help establishing efficient monitoring programmes including risk assessments of *Ae*. *albopictus* [16]. We here provide maps on the potentially most limiting climatic factor for *Ae*. *albopictus* in Europe under current and future conditions. As *Ae*. *albopictus* is one of the most well-studied mosquito species it is a suitable model species for evaluating this maximum entropy limiting factor mapping approach. In order to evaluate this approach we first accounted for the robustness against variable choice by comparing the modelling results based on two different environmental variable sets. Secondly, we compared our results with the commonly suggested thresholds for an establishment of *Ae*. *albopictus* in Europe that are derived from the observed geographical distribution and based on climate chamber experiments.

## Material and Methods

### Species distribution modelling

The basic approach of species distribution models (SDMs), often also called ecological niche models (ENMs), is to correlate species’ presences or absences with environmental variables prevailing in the respective locations in order to project the potential distribution of a species under current and future climatic conditions. However, these projections can be based on many different statistical algorithms; all aiming at estimating this species-environment-relationship best. Here, the maximum entropy approach, implemented in the software Maxent (v. 3.3.3k [17,18]) was chosen. The Maxent approach is one of the most commonly employed algorithms to model species potential ranges (e.g. [19], it scores well in comparative studies (e.g. [20]) and the modelled niche function is relatively easy to handle from a mathematically point of view (cf. [21]). According to [22], the maximum entropy approach is relatively insensitive to spatial errors associated with location data, requires few locations to construct useful models, and performs better than other presence only modelling algorithms. These may be especially important issues considering *Ae*. *albopictus* as non-native invasive species in Europe. We used the default setting, but only linear, quadratic and product features (cf. [21]). The modelling results were displayed in maps built using ESRI ArcGIS (Release 10.3).

### Occurrence data and environmental variables used for modelling

We used 336 occurrence records for *Ae*. *albopictus* in Europe (within the extent of the study area: 10°W– 45°E; 35°N– 79°N), taken from [23] and [6] (see Supporting information). The records were adjusted to the raster of the environmental variables with a spatial resolution of 5 arc minutes (ca. 10 km * 10 km). We modelled the ecological niche of *Ae*. *albopictus* based on (I) annual mean temperature, mean temperature of the warmest quarter, temperature of the coldest quarter, precipitation of warmest quarter and annual precipitation (model 1) and (II) mean January temperature (see [11]), mean summer temperature (June, July and August), precipitation in spring (March, April and May), precipitation in summer (June, July and August) and the number of days with a day length of more than 13 hours (model 2). The latter variable is called “photoperiod” and was calculated using the R package geosphere version 1.4–3. The climatic variables considering current conditions were obtained from Worldclim (www.worldclim.org). Data on projected future climatic conditions were taken from CIAT (http://www.ccafs-climate.org/data/) based on the global circulation model CSIRO-Mk3.6.0. The combination of predictor variables in model 1 is similar to a combination that has proven to be successful in previous studies using correlative approaches [7,6] but we decided to consider temperature annual range instead of altitude, as altitude is regarded as a proxy for temperature. Although Maxent is considered relatively robust against collinear variables, (i.e. collinearity does not affect the performance of Maxent) we tried to exclude strongly inter-correlated predictor variables as inter-correlation can impair the interpretation of variable influence [24]. In model 2, predictor variables were aligned to variables of process-based models (e.g. [1,11]) which account for inter-correlation of variables.

When transforming the continuous values between 0 and 1 for the modelled habitat suitability under current climate conditions into dichotomous modelling results (i.e. habitat suitable or not suitable) we applied a threshold that minimized the difference between sensitivity and specificity as optimization criterion [25].

### Specification of the most limiting factor

In order to determine the most limiting variable, we used the Maxent limiting factor mapping tool described in [14]. This feature identifies the variable which influences the projected habitat suitability the most for each pixel of the study area. The model is run N times, where N is the number of considered environmental variables. In each run one predictor variable is set to its mean value across the occurrence data, assuming this is an optimal value considering this variable. Changing this variable from its recorded value to this assumed optimal value may increase the modelled habitat suitability at a selected site. If changing a predictor variable from its recorded value at the selected site to its mean value across the occurrence records increases the modelled habitat suitability at this point more than it does for another variable, this variable will be considered to be the most limiting factor at that point.

We mapped the most limiting factors for *Ae*. *albopictus* for Europe under current and projected future climatic conditions for both models (model 1 and model 2).

### Further analysis

As mentioned above, a successful establishment of *Ae*. *albopictus* in Europe requires several thresholds of different climatic parameters to be exceeded. According to the European Centre for Disease Prevention and Control (ECDC, ecdc.europa.eu), an annual precipitation of at least 500 mm is thought to be required to ensure the availability of small aquatic habitats for egg deposition, a winter temperature of above 0°C to ensure egg survival, and a summer temperature of above 11°C seems necessary for adult survival and activity. For latest review see [9]. We displayed the areas in Europe matching the establishment criteria and compared those with the results of the correlative species distribution models. The maps with areas matching the establishment criteria may help to interpret the results of the limiting factor modelling in order to assess our results.

## Results

### Habitat suitability maps (Fig 1)

**Fig 1:**
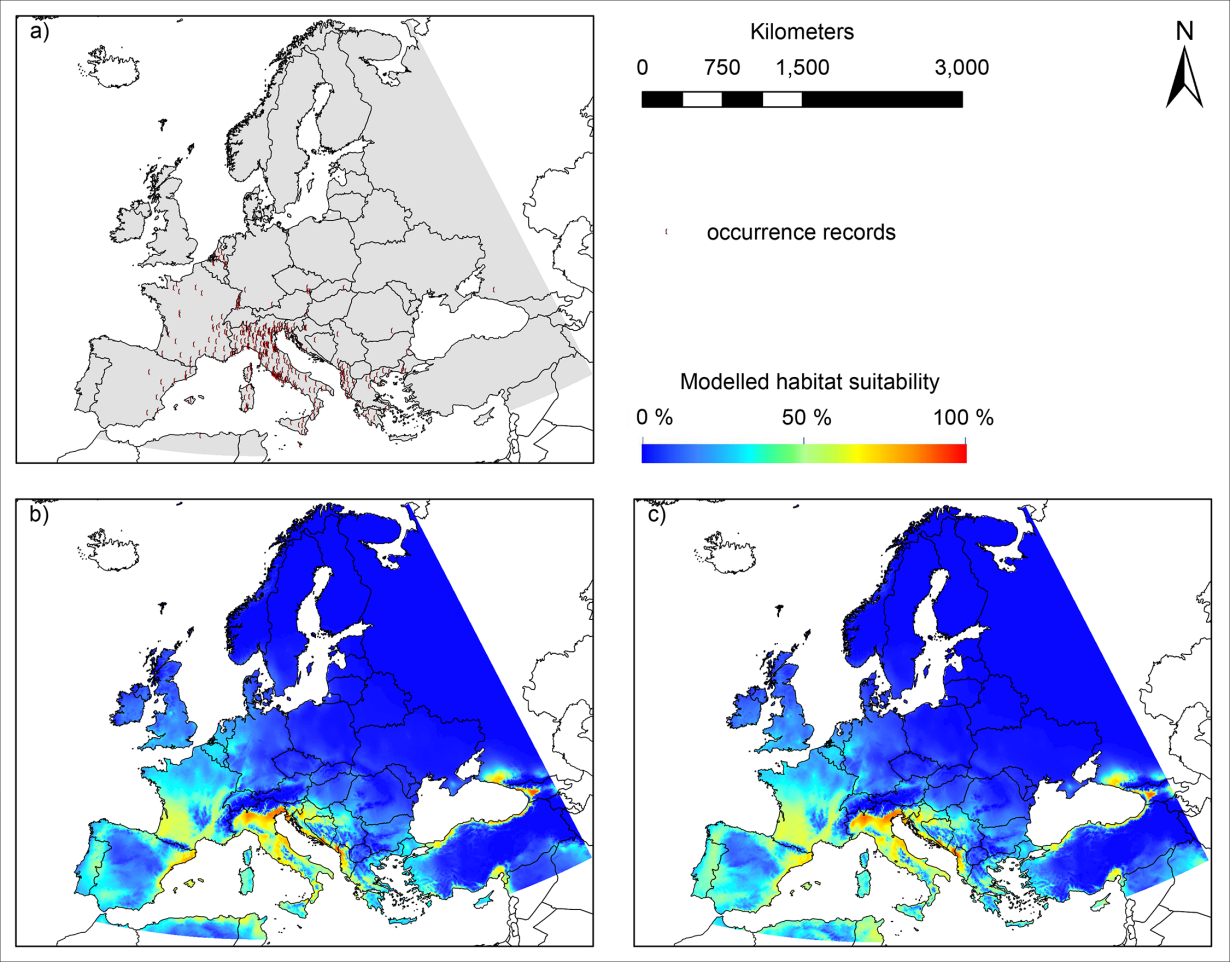
Observed distribution and modelled habitat suitability for *Ae*. *albopictus* under current climatic conditions in Europe. A) Occurrence records for *Ae*. *albopictus* used for modelling B) modelled habitat suitability (model 1) under consideration of mean temperature of coldest quarter, mean temperature of warmest quarter, annual mean temperature, precipitation of warmest quarter and annual precipitation and C) modelled habitat suitability (model 2) under consideration of mean temperature in January, mean temperature in summer, photoperiod, precipitation in spring and precipitation in summer. Geographical projection: Europe Albers Equal Area Conic. Maps were built using ESRI ArcGIS.

According to our modelling results, suitable habitat conditions for *Ae*. *albopictus* can be found especially in Southern Europe: Portugal and adjacent areas in Western Spain, large parts of France, especially the Western of the country and the Rhône valley, Italy and parts of the Balkan (Fig 1B and 1C). The models based on the two different sets of environmental variables show a high agreement in terms of the pattern of modelled habitat suitability. In species distribution modelling the area under the receiver operating characteristic curve (AUC value) is a commonly used measure to compare the performance of different models. Using a replication of 100, both models performed similarly, with model 1 (Fig 1B) and model 2 (Fig 1C) having AUC values of 0.925 +/- 0.053 SD and 0.923 +/-0.052 SD, respectively. In both models, winter and summer related temperatures were the variables that overall contributed most to the models according to the permutation importance criteria, with 61.7% and 65.7% for winter related variables and 21.2% and 17.8% for summer related variables (Table 1).

**Table 1:** Permutation importance as a measure of relative contributions of the environmental variables to the Maxent models.

**Variable**	**Permutation importance (%)**
**Model 1**	
**mean temperature of coldest quarter**	61.7
**mean temperature of warmest quarter**	21.2
**precipitation of warmest quarter**	10.8
**annual precipitation**	6.3
**annual mean temperature**	0.0
**Model 2**	
**mean temperature in January**	65.7
**mean temperature in summer**	17.8
**precipitation in summer**	11.4
**precipitation in spring**	5.0
**photoperiod**	0.0

### The most limiting factors under current climatic conditions (Fig 2)

**Fig 2:**
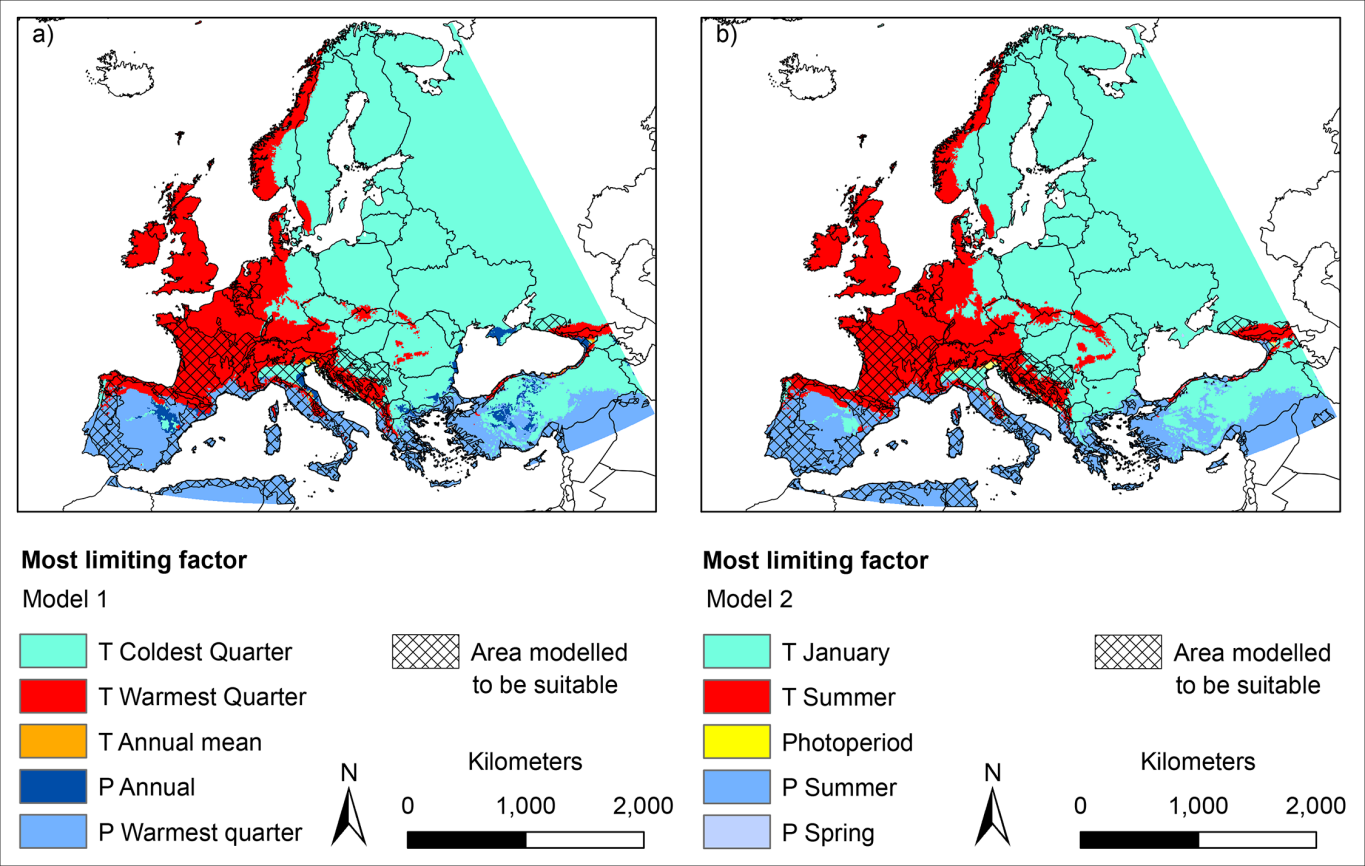
Limiting factors under current climatic conditions based on two Maxent models A) model 1 under consideration of mean temperature of coldest quarter, mean temperature of warmest quarter, annual mean temperature, annual precipitation and precipitation of warmest quarter and B) model 2 under consideration of mean temperature in January, mean temperature in summer, photoperiod, precipitation in spring and precipitation in summer. For any point, the limiting factor is the variable whose value influenced the model prediction the most. Cross-hatched areas indicate area with modelled habitat suitability for *Ae*. *albopictus* (dichotomous modelling results). Geographical projection: Europe Albers Equal Area Conic. Maps were built using ESRI ArcGIS.

According to our modelling results with two different sets of environmental variables, *Ae*. *albopictus* is, under current climatic conditions, mainly restricted to the Mediterranean region, the west of the Iberian Peninsula and Western France (Fig 2, hatched area). Cold temperatures in winter have been identified as the most limiting factor for the mosquito in Eastern Europe in both models, with the mean temperature of the coldest quarter and the mean temperature in January as the most limiting factors in model 1 and model 2, respectively. For Central Europe, summer temperatures, i.e. mean temperature of warmest quarter in model 1 and temperatures in June, July and August in model 2, were the most limiting factors. In the southern parts of Europe precipitation during summer has been identified as the most limiting factor in both models.

### Threshold for the successful establishment in Europe under current climatic conditions (Fig 3)

**Fig 3:**
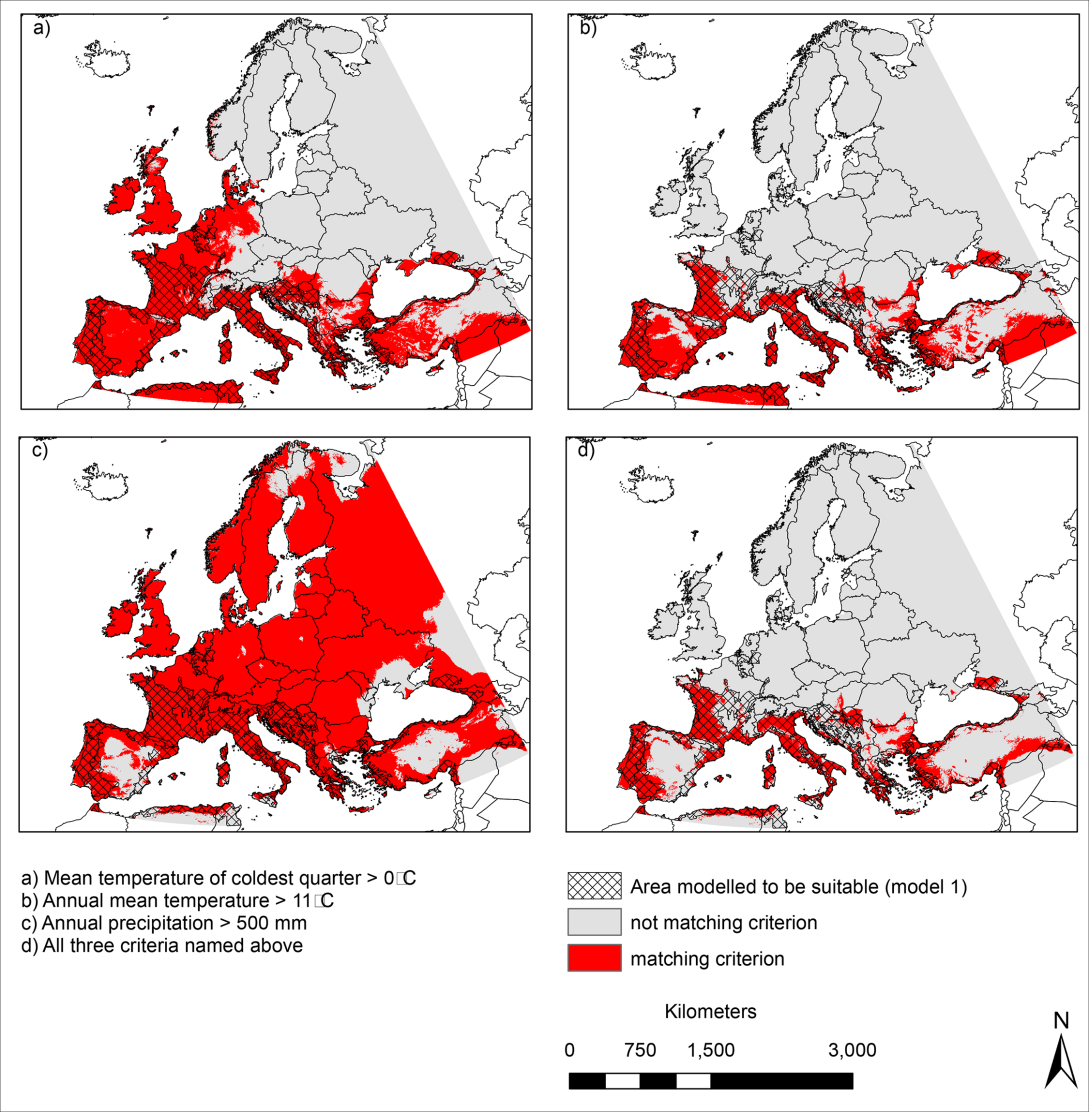
Areas that match the establishment criteria (according to Medlock et al. 2015) under current climatic conditions: A) area with a mean temperature of the coldest quarter above 0°C, B) area with an annual mean temperature of > 11°C, C) area with an annual precipitation of > 500 mm, D) area for which criteria A), B) and C) are fulfilled. The cross-hatched area indicates suitable habitat conditions for *Ae*. *albopictus* according to the dichotomous modelling results based on model 1. Maps were built using ESRI ArcGIS.

In terms of the establishment thresholds (coldest quarter of > 0°C, annual mean temperature of > 11°C and annual precipitation of > 500 mm), Northern and Eastern Europe were modelled unsuitable for an establishment of *Ae*. *albopictus* due to winter temperatures and annual mean temperatures below the respective thresholds (Fig 3A and 3B). Considering the annual precipitation criterion, only Central Spain, parts of Scandinavia, Turkey and the southernmost boundary of the study area seem unsuitable for an establishment of *Ae*. *albopictus* (Fig 3C). When all three thresholds were applied together, the area considered to be suitable for an establishment of *Ae*. *albopictus* greatly overlapped with the area modelled to be suitable according to the dichotomous modelling results of the species distribution modelling (model 1 –hatched area in Fig 3).

### The most limiting factors under future climatic conditions (Figs 4 and 5)

**Fig 4:**
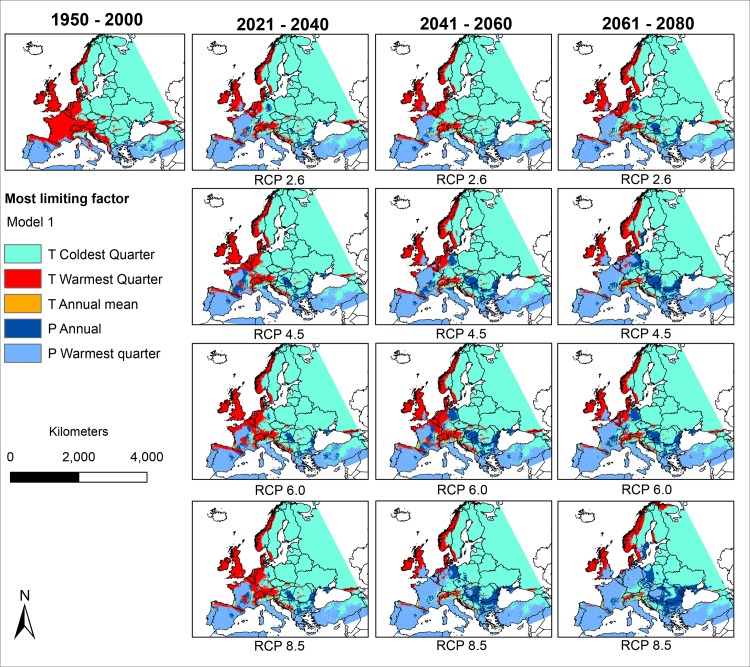
The most limiting factors for the potential distribution of *Ae*. *albopictus* based on model 1 under consideration of mean temperature of coldest quarter, mean temperature of warmest quarter, annual mean temperature, annual precipitation and precipitation of warmest quarter under current and projected future climatic conditions according to the IPCC AR5 data GCM: CSIRO. For any point, the limiting factor is the variable whose value influenced the model prediction the most. Geographical projection: Europe Albers Equal Area Conic. Maps were built using ESRI ArcGIS.

**Fig 5:**
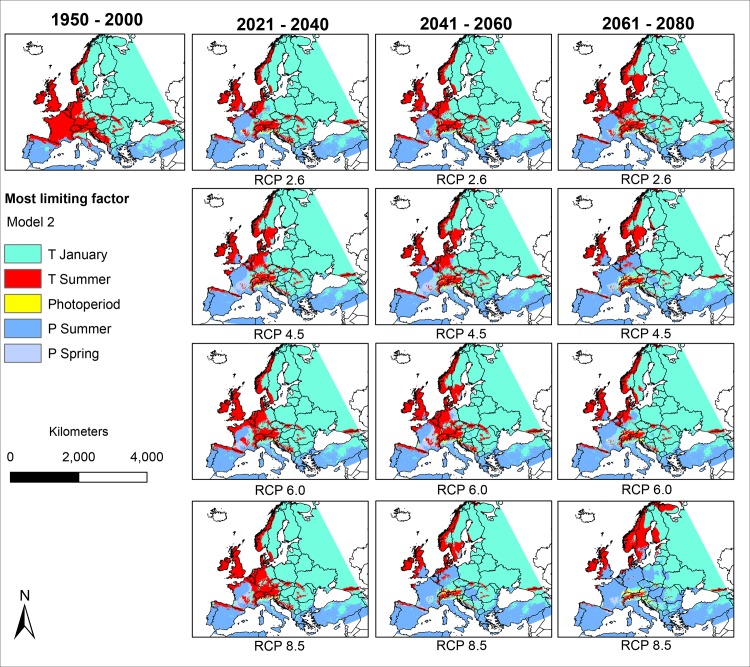
The most limiting factors for the potential distribution of *Ae*. *albopictus* based on model 2 under consideration of mean temperature in January, mean temperature in summer, photoperiod, precipitation in spring and precipitation in summer under current and projected future climatic conditions according to the IPCC AR5 data GCM: CSIRO. For any point, the limiting factor is the variable whose value influences the model prediction the most. Geographical projection: Europe Albers Equal Area Conic. Maps were built using ESRI ArcGIS.

Under projected future climatic conditions, precipitation was modelled to become more important as a limiting factor for *Ae*. *albopictus*, as can be seen in the increase of blue area in Figs 4 and 5. The areas in which precipitation was modelled to be the most limiting factor were modelled to expand northwards under future climatic conditions. In contrast, areas where summer temperature was modelled to be the most limiting factor were modelled to get smaller under future climatic conditions or shift northwards.

## Discussion

Here, habitat suitability for *Ae*. *albopictus* in Europe was modelled using two sets of environmental variables that are considered to be relevant for the species within its non-native range. The first set arose from previous correlative studies [7,6], the second one is commonly used in process-based models on *Ae*. *albopictus* (e.g. [1,11]). Despite the different variable sets, however, both models highly corresponded in the pattern of modelled habitat suitability and the high AUC values of over 0.9. This emphasizes the robustness of the estimation. Both models showed similar projections for the habitat suitability for *Ae*. *albopictus* under current climatic conditions in Europe with highest suitability in Southern Europe. In contrast, habitat conditions in Central and Eastern Europe were modelled to be unsuitable for *Ae*. *albopictus*. Whereas both models are generally in good accordance with observed occurrences of *Ae*. *albopictus* in Europe (Fig 1A), as well as the modelling results of previous studies (e.g. [7,1]), for some regions modelled habitat suitability and occurrences did not match, most obviously for parts in Spain and France. This might be explained by a potential dispersal limitation for the species, i.e. despite climatically suitable conditions in these regions the species has not been introduced in these regions or did not continue its spread from other areas where it has already established.

Apart from estimating the habitat suitability for *Ae*. *albopictus*, we applied the Maxent limiting factor mapping tool described in [14] to identify key climatic factors for the potential distribution of the mosquito species in Europe. Our modelling approach identified three key limiting factors for *Ae*. *albopictus* in Europe: winter temperature (i.e. temperature of coldest quarter in model 1 and mean temperature in January in model 2) in Eastern Europe, summer temperature (i.e. temperature of warmest quarter in model 1 and temperature in June, July and August in model 2) in Central Europe and summer precipitation (i.e. precipitation of the warmest quarter in model 1 and precipitation in June, July and August in model 2) in Southern Europe. This is in good accordance with the limits commonly put forward as thresholds for a successful establishment in Europe, i.e. January temperature > 0°C, annual mean temperature > 11°C and annual precipitation > 500 mm [9]. According to [11] the 0°C January-Isotherm and the 11°C annual mean temperature isotherm correspond best to the northern limits for the observed distribution of *Ae*. *albopictus* in Northern Italy. This corresponds well with our results: The area modelled to be suitable for *Ae*. *albopictus* widely matches the area with an annual mean temperature above 11°C and a January mean temperature of above 0°C. The importance of these two factors was also supported by their high relative contributions to the Maxent models (Table 1). The area matching all three thresholds corresponds very well to the area with modelled suitable habitat conditions according to our correlative modelling approach (model 1, dichotomous results). There is a good accordance of the results of our correlative approach (based on species occurrence records) and those of the process-based approach (based on thresholds representing species ecological requirements that are rarely available and only for well-studied species). To summarize, the robustness of the variable choice, the good accordance of the modelled habitat suitability area with the area matching all commonly supposed establishment thresholds and thus the plausibility of our results against the background of the knowledge about the species’ ecology suggest a good performance and reliability of the approach.

Low temperatures play only a minor role in its native range, where *Ae*. *albopictus* is active throughout the year with no need for overwintering stages [9]. In temperate regions, however, several authors have suggested that low temperatures might inhibit the survival of individuals and thus, restrict a successful establishment of *Ae*. *albopictus* in Europe (e.g. [2,1]). Despite the fact that populations of *Ae*. *albopictus* in temperate regions are supposed to be adapted to colder winter temperatures and able to undergo long periods of diapause [16], temperatures below a certain minimum threshold would be fatal, for adult but also for egg survival. The latter has been tested in climate chambers but not in the field so far [5]. The continental climate of Eastern Europe (Central Poland and eastwards) is characterized by a large amplitude of the annual temperature cycle, and thus, by comparably low temperatures in winter. According to our results, these temperatures, i.e. the temperature of the coldest quarter (model 1) and the temperature in January (model 2), respectively, were the most limiting factors in Eastern Europe. Due to a milder oceanic climate, winter temperatures are usually much higher in Western Europe compared to Eastern Europe and thus, generally of less consequence to *Ae*. *albopictus*. In contrast, here, summer temperatures were modelled to be the most limiting factor under current climate conditions, more specifically temperature of the warmest quarter (model 1) and temperature in summer (model 2). This might be explained by the fact that temperatures of 25–30°C, which are supposed to be optimal for the development of *Ae*. *albopictus* [9], lie well above the average summer temperatures reached within those regions today.

Apart from temperature as the limiting factor, low precipitation, especially in summer, may limit the availability of breeding places as the species requires small aquatic habitats for egg deposition [1]. According to our modelling results, the absence of suitable habitat conditions for *Ae*. *albopictus* in Central Spain can be ascribed to low precipitation, more specifically precipitation in summer. This factor was identified as the most limiting in both models in Southern Europe. Higher precipitation rates are generally assumed to enhance the availability of suitable breeding habitats. [9] supposing an overall thresholds of at least 500 mm annual precipitation necessary for egg deposition. However, the role of precipitation with regard to the potential distribution of *Ae*. *albopictus* has been discussed controversially (e.g. [9,26,27]). High amounts of precipitation certainly ensure the availability of small aquatic habitats that can be used for egg deposition. In arid regions this is partially compensated by human water supply, especially in urban areas. Some authors suggest that human water supply is even more important than precipitation. Despite this, lower precipitation during summer could become a limiting factor for *Ae*. *albopictus* in the following decades in Southern Europe. Here, summer precipitation was identified as the most limiting factor in the southern parts of the study area. The Aegean region as well as the southernmost boundary of the study area are–except for parts of Scandinavia for which winter temperature is modelled the most limiting factor—the only regions within the study area where the annual precipitation is lower than the 500 mm thresholds (Fig 3C). Overall, temperature shows a stronger influence on the geographical range of *Ae*. *albopictus* in Europe than precipitation [26].

Continuous trade and global tourism will promote accidental introduction of individuals of *Ae*. *albopictus* into Europe and also beyond regions where they have already established. *Ae*. *albopictus* is a competent vector of several diseases, especially dengue and chikungunya viruses. Due to this medical relevance, there is a special interest in assessing the invasive potential and in estimating the potential future spread of the species. According to the models based on future climatic conditions, low winter temperature in Eastern Europe will remain the most limiting factor for *Ae*. *albopictus*. However, summer temperature seems to become replaced by annual precipitation and summer precipitation respectively as most limiting factors in Central Europe in the future. The latter would also fit latest climate change projections for these regions, with generally higher temperatures and less precipitation during summer (IPCC, [28]).

As the species has been subject to numerous studies before and its ecology is well-known, we were able to evaluate the approach by [14] in order to identify the factors most limiting for the modelled habitat suitability and consequently for establishment of the species. The investigation of limiting factors did additionally provide valuable insights into the major drivers of current and future distribution of *Ae*. *albopictus*. Results generated here could be used to help coordinate monitoring programmes, especially in regions where individuals are currently absent but habitat conditions were modelled to be suitable for *Ae*. *albopictus* under future climate change. Based on our results we suggest that the approach taken here might be similarly applicable to other invasive species.

## Supporting Information

S1 Dataset Occurrence data for *Aedes albopictus* used for modelling. The records were adjusted to the raster of the environmental variables with a spatial resolution of 5 arc minutes.
(CSV)
